# Reproduction of the human relevant potency threshold (HRPT) for estrogen receptor alpha agonism in an inference performance screen for ICCVAM’s regulatory scientific confidence framework

**DOI:** 10.3389/ftox.2025.1657708

**Published:** 2025-10-08

**Authors:** Jessica Ryman, Richard A. Becker

**Affiliations:** American Chemistry Council, Washington, DC, United States

**Keywords:** new approach methods, interagency coordinating committee for the validation of alternative methods, scientific confidence framework, endocrine disruptors, Human Relevant Potency Threshold, estrogen receptor alpha

## Abstract

Scientific confidence frameworks (SCFs) are alternatives to traditional validation for new approach methodologies (NAMs). The SCFs adapted by the Interagency Coordinating Committee for the Validation of Alternative Methods (ICCVAM) and the American Chemistry Council (ACC) both address inference performance—the ability of NAMs to predict or inform the biological effect of interest. Inference performance is a distinct evaluation procedure in ACC’s SCF but is blended into several steps of ICCVAM’s SCF. Here, we first reproduce the previously derived human relevant potency threshold (HRPT) for the estrogen receptor alpha (ERα) agonism of Borgert et al. (2018) using guideline and guideline-like studies; we found that a HRPT of 1 to 10^–1^ positively and consistently predicted clinical endometrial and endocervical effects. We next mapped inference performance to ICCVAM’s SCF and found that it can be used as an effective initial screen prior to performing more detailed characterizations in their SCF. We first conclude that a HRPT for ERα agonism of 10^–2^ to 10^–4^ is a health-protective NAM based on an established mode of action that could potentially be used in early screening, much like the threshold of toxicological concern. We then conclude that inference performance is a core requirement for SCFs.

## 1 Introduction

The United States Environmental Protection Agency’s (EPA’s) “Strategic Plan to Promote the Development and Implementation of Alternative Test Methods” defines a new approach methodology (NAM) as “…any technology, methodology, approach, or combination thereof that can be used to provide information on chemical hazard and risk assessment that avoids the use of intact animals” (US EPA, 2018). While this definition does not explicitly include reducing or refining the use of vertebrate animals, these are components of this plan. The plan was required due to the 2016 amendments to the *Toxic Substances Control Act* (TSCA) as a result of the *Frank R. Lautenberg Chemical Safety for the 21st Century Act*. The amended TSCA is one of an increasing number of regulatory drivers for the adoption of NAMs in the United States and globally.

For the last two decades, the Organization for Economic Co-operation and Development (OECD, 2005) has governed the validation NAMs for regulatory use for environmental chemicals. It includes formal validation in multiple laboratories, also known as “ring trials.” However, the time- and resource-intensive nature of formal validation via ring trials has not facilitated the timely regulatory acceptance of NAMs or kept pace with increasing regulatory mandates to reduce animal testing while increasing chemical knowledge, nor has formal validation kept pace with the rapid technological development of NAMs. This has led to increasing calls for replacing formal with fit-for-purpose validation. However, the term “fit-for-purpose validation” has not been defined or operationalized. It has also led to concerns of a reproducibility crisis if ring trials were abandoned (Jacobs et al., 2024).

A decade ago, the concept of a scientific confidence framework (SCF) began to emerge in work done by the American Chemistry Council’s (ACC’s) Long Range Research Initiative (LRI) as a tool for operationalizing fit-for-purpose validation. The elements of the ACC’s SCF emerged organically, first in the transparent evaluation of *in vitro* (Tox21) assays and the associated prediction models for estrogen, androgen, thyroid, and steroidogenesis (EATS) outcomes in the EPA’s Endocrine Disruption Screening Program (EDSP) (Cox et al., 2014) and then when evaluating adverse outcome pathways (AOPs) (Patlewicz et al., 2015). Together, the learnings and approaches from these investigations formed the basis for a formal articulation of ACC’s SCF for the use of NAMs in general (Becker and Ryman-Rasmussen, 2023; Ryman-Rasmussen and Becker, 2023; Ryman-Rasmussen and Becker, 2024; Ryman-Rasmussen, 2023; Ryman and Becker, 2024).

**FIGURE 1:**
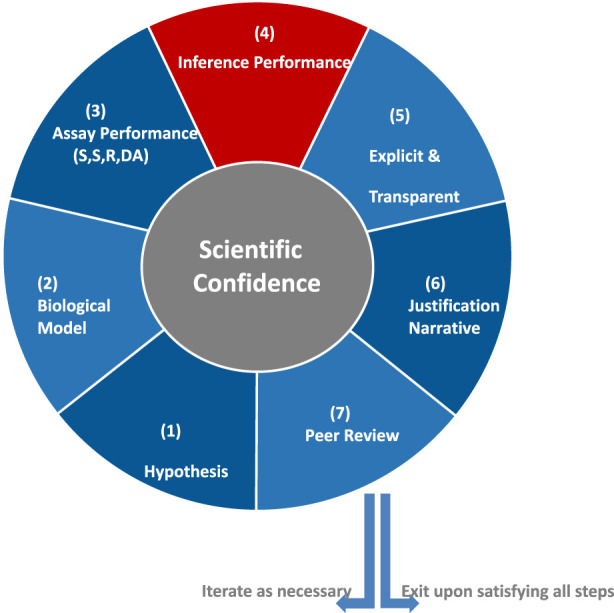
ACC’s Scientific Confidence Framework displayed as a cycle that can be exited upon satisfying all seven of the steps. However, the cyclic framework can be iterated as necessary if steps are not satisfied or as new data are available.

ACC’s SCF comprises seven steps (Figure 1). Step 1 (Hypothesis) consists of problem formulation and articulation of the hypothesis for how the NAM can be used to provide actionable information for a specific decision context. Step 2 (Biological Model) considers the biological relevance and plausibility of the NAM. Step 3 (Assay Performance) documents the sensitivity, specificity, reliability, and domain of applicability of the NAM. Step 4 (Inference Performance) assesses whether the NAM endpoint predicts the outcome of interest. The ability of the response in the NAM to predict the response of interest is also called the “response–response relationship.” Such a NAM could have a mechanistic or non-mechanistic basis. As described in the next paragraph, inference performance is a discreet step that sets this SCF apart from ICCVAM’s SCF. Step 5 (Explicit and Transparent Dissemination) requires dissemination of the data, inference models, and other information that supports independent replication. Step 6 (Justification Narrative) is the rationale that makes the case that there is/is not sufficient scientific confidence in the NAM to support the specific application of interest. Step 7 (Peer Review) requires verification through independent scientific peer review.

An SCF similar to ACC’s was published by van der Zalm et al. (2022). This similarity indicates consensus in SCFs that has emerged independently. That said, there are some differences, with van der Zalm et al.’s key differences being that ACC’s discrete Step 4 (Inference Performance) is woven into technical characterization related to accuracy, and there is no separate justification narrative. Van der Zalm et al. (2022) strongly influenced the development of the Interagency Coordinating Committee on the Validation of Alternative Methods (ICCVAM)’s 2024 “Validation Qualification, and Regulatory Acceptance of New Approach Methodologies,” which is essentially an SCF (Interagency Coordinating Committee on the Validation of Alternative Methods (ICCVAM) 2024). ICCVAM’s SCF also shares similarities to that of ACC. However, ACC’s discrete Step 4 (Inference Performance, or prediction of the response–response relationship) is blended over several steps of ICCVAM’s SCF when the NAM is investigated to determine its ability to inform the response in humans or environmental organisms of interest.

Screening for endocrine disruption (which is a mode of action and not an adverse effect *per se*) has been an early area in which NAMs have been leveraged in tiered testing strategies. The EPA’s Endocrine Disruptor Screening Program (EDSP) utilizes NAMs to inform subsequent steps for identifying and characterizing hazards observable in intact organisms that result from an endocrine mode of action (MOA). Estrogen receptor transcriptional activation is measured *in vitro* in the exquisitely sensitive estrogen receptor transcriptional activation (ERTA) assay. The ability to elicit an estrogen-sensitive effect in a whole organism is measured *in vivo* in the rodent uterotrophic assay, where the uterus—an already estrogen-sensitive tissue—is further sensitized by using immature or ovariectomized animals. Together, these *in vitro* and *in vivo* assays are a measure of the mechanistic potency of estrogen receptor alpha (ERα) affinity and activity that have been harnessed by the pharmaceutical industry to assist in drug development as well as by the EDSP (Borgert et al., 2018). Importantly, the promiscuity of the ERα due to its flexible binding pocket (Ng et al., 2014), coupled with these highly sensitive *in vitro* and *in vivo* endpoints, allows low potency substances to be detected that may not be active in humans. In order to identify human-relevant potencies, the mechanistic potencies at these endpoints for various ERα agonists relative to estradiol (E2) or pharmaceutical ERα agonists (e.g., ethinyl estradiol (EE2) or diethylstilbestrol (DES)) were compared with clinically observable estrogenic effects in humans to develop the human relevant potency threshold (HRPT). A relative potency of 1 × 10^−4^ was determined to be a conservative estimate of a “…potency threshold below which adverse effects in humans are unlikely.”

The HRPT for ERα agonism is arguably an NAM based on an established MOA and could be used in a tiered approach much like the threshold of toxicological concern (TTC, Simon et al., 2024). While the rodent uterotrophic assay may still be necessary, the HRPT nonetheless may reduce the need for animals in subsequent testing (such as in screening out the need for developmental/reproductive toxicity studies) if the potency is below the threshold for mechanistic potency. As such, the HRPT for ERα agonism would be a scientifically defensible and health-protective stopping point for further *in vivo* testing.

Here, we first reproduce the analysis of Borgert et al. (2018) utilizing regulatory guideline and guideline-like studies (GLL). Based on this, we hypothesize that substances with relative functional potency within 1 × 10^−4^ for ethinyl estradiol (EE2)-mediated transcription and uterotrophic effects at ERα would predict increased clinically observable increases in uterine endometrial and/or endocervical effects to include endometrial hyperplasia and adenomas and carcinomas (cancer). Unlike Borgert et al. (2018), we narrowed human clinical effects in the endometrium and cervix to conform more closely to uterotrophic effects in rodents. This choice is due to the known ability for certain ERα agonists to exert tissue-specific effects as selective estrogen receptor modifiers (SERMs). This hypothesis corresponds to Step 4 (Inference Performance) in ACC’s SCF. We next hypothesize that by satisfying Step 4 of ACC’s SCF such that the response in the NAM was predictive of the response in humans, several elements of ICCVAM’s SCF would be satisfied. If proven true, this would indicate that Step 4 of ACC’s SCF could serve as an initial screen to indicate the suitability of an NAM prior to more in-depth analysis via ICCVAM’s SCF.

## 2 Materials and methods

### 2.1 ERTA and rodent uterotrophic data

EC_50_ values from Table 1 of the OECD Test Guideline (TG) No. 455 “Performance-Based Test Guideline for Stably Transfected Transactivation *In Vitro* Assays to Detect Estrogen Receptor Agonists and Antagonists” contains results from the STTA and VM7Luc ER TA assays for ER agonists or negatives. For the STTA assay, the PC_10_ and PC_50_ for E2 were estimated only as < 1.00 × 10^−11^ and not actually identified. Therefore, we utilized the identified EC_50_ values from the VM7Luc ER TA assay for ERα agonists for which rodent uterotrophic and clinical data are available.

**TABLE 1:** Comparison of ICCVAM’s SCF with ACC’s Step 4, inference performance (i.e., the extent to which the NAM response predicts the response of interest).

ICCVAM’s SCF	ACC’s SCF step 4
1. Context of use, to include regulatory purpose (if applicable) (e.g., screening and prioritization)	Informs context of use by providing information on degree of predictiveness, which will determine whether NAM is suitable for screening level, supporting information, or stand-alone status.
2. Biological Relevance	Informs biological relevance by requiring an understanding of the strengths and limitations of the NAM with respect to the response of interest.
2.1 Mechanistic Understanding	
2.2 References Compounds	
2.3 Comparison to Existing Laboratory Animal Methods	
3. Technical Characterization	Informs the necessary technical elements and performance standards and the limits of use.
3.1 Incorporation of Selected Quality Tools	
3.2 Best Practices for Quality Control	
3.2.1 Relevant Information for Cell/Tissue Methods	
3.2.2 Assessing the Analytical Method Used in the NAM	
3.2.3 Assessing Accuracy and/or Concordance of the NAM with Performance Standards	
3.2.4 Standard Operating Procedures and Method Details	
3.3 Documentation	
3.3.1 Test Substance Identity and Purity	
3.3.2 Method Development	
3.3.3 Endpoint and Parameter Measurements	
3.3.4 Limits of Use	
3.3.5 Well-Defined Endpoint	
3.3.6 Building a Statistical Model	
3.3.7 Reproducibility of the Assay Results	
3.3.8 Data Interpretation Procedure	
4. Data Integrity	Incorporated in Steps 3 and 5 of the ACC Scientific Confidence Framework.
5. Information Transparency	Informs transparency regarding NAM’s relevance to context of use.
6. Independent Review	Incorporated in step 7 of the ACC Scientific Confidence Framework.

For rodent uterotrophic assay data, Kleinstreuer et al. (2016) identified GLL studies and entered them into a curated database (UTDB). GLL studies active in this assay were first matched to ERα agonists in Table 1 of OECD TG 455. EC_50_s were visually estimated from already published dose-response curves or fit from published mean, SD, and n data in GraphPad Prism v.10.4.1. In Prism, a least-squares fit for agonist vs response, variable slope (four parameters) was utilized. Results were expressed as relative potency to EE2. EE2, not the endogenous agonist E2, was selected because the former is more commonly used as the reference chemical.

### 2.2 Human clinical correlates in the endometrium

Due to the ability of ERα agonists to exert selective effects in different tissues, human clinical correlates were limited to those relevant to the rodent uterotrophic assay. This assay removes both the uterus and cervix prior to weighing (OECD, 2007). Increases in weight result from fluid imbibition and/or hypertrophy and/or hyperplasia (Marty and O’Connor, 2014). We selected clinical correlates in humans for this effect of endometrial and endocervical thickening and hyperplasia. Due to concerns for carcinogenicity from sustained ERα activation, we also included endometrial and cervical cancer.

The ERα agonists for which both OECD TG 455 and UTDB were available that are endogenous agonists or that have (or had) clinical or dietary exposures are E2, 17α-E2, EE2, DES, daidzein, estrone (E1), genistein, hexestrol, and reserpine. Of these, diadzein and reserpine had conflicting data in the UTDB. A positive effect for diadzein, hexestrol, and reserpine was only observed at one dose and therefore not amenable to EC_50_ derivation. Therefore, E1, E2, 17α-E2, EE2, DES, and genistein were carried forward for analysis.

The proliferative histopathological effects of endogenous E2 and exogenous E2 and EE2 on the endometrium are well characterized (Deligdisch, 2000). Prenatal exposure to maternally therapeutic levels of DES is strongly associated with the rare lesion of adenocarcinoma of the cervix, is generally regarded as causal, and was first reported in young persons assigned female at birth (AFAB) by Herbst et al. (1971). For phytoestrogens, a European Food Safety Authority on Food Additives and Nutrient Sources review (EFSA ANS Panel, 2015) of peri- and menopausal persons taking 30–150 mg/day isoflavone supplements was first consulted for endometrial thickening and histopathological effects for genistein. This was followed by additional literature searches for clinical studies in PubMed.com for endometrial safety studies on isoflavones containing genistein, for E1 and 17α-E2, which are endogenous estrogens, and for “isoflavone” and “endometrium” and “hyperplasia” and “endometrial safety”. E1 and 17α-E2 are endogenous estrogens that are also therapeutically prescribed for managing menopausal symptoms and hair loss, respectively. As such, the literature and drug safety information were searched for indications of proliferative effects on the endometrial and endocervical epithelium. The compiled data were displayed in tabular format.

### 2.3 Inference performance evaluation (ACC’s SCF step 4)

ICCVAM’s SCF and ACC’s Step 4 (the extent to which the NAM’s response predicts the response of interest) were compared in Table 1. The elements of the two SCFs are similar, with the exception that the ACC SCF requires a specific evaluation of the performance of the qualitative or quantitative inference model.

The relative functional potencies of the ERTA and uterotrophic data (relative to E2 or EE2) were compared to the clinical data to determine whether these predicted clinical effects were within a relative potency equal to or greater than 1 × 10^−4^. These are compared in Table 2.

**TABLE 2:** Comparison of relative mechanistic potencies (ERαTA + rodent uterotrophic assay) to clinical endometrial/cervical effects.

	Mechanistic potency		Human clinical effects
	OECD ERαTA Relative (to E2)^a^	Uterotrophic Assay (GLL-UTDB) Relative Potency (to EE2)^c^	Endometrial hyperplasia Endometrial/cervical adenoma or carcinoma
E2	1.0E+00 (1.0E+00)^b^	9.0 E-01 (ovx mouse, ip^d^) (NA)^b^	YES Hyperplasia (endogenous) Carcinoma (exogenous)
EE2	7.7E-01 (1.1E+00 to 5.7E+00)^b^	1.0E+00 (immature/ovx rat sc) (NA)^b^ 1.0E+00 (immature/ovx rat po) (NA)^b^	YES Hyperplasia Carcinoma (exogenous)
DES	1.7E-01 (2.5-01 – 8.0E+00)^b^	7.8E-01 (immature rat, sc) (NA)^b^ 3.0E-01 (immature rat, po) (9.3E-01, 6.8E-01) (immature rat, po)^b^	YES Cervical carcinoma (exogenous)
Estrone (E1)	2.4E-02 (1.0E-02 to 7.5E-02)^b^	1.7E-01 (ovx mouse, ip^d^) (1.0E-03) (immature rat, sc)^b^	NE (endogenous and exogenous, inactive/less active precursor converted to E2 in endometrium)
17-alpha estradiol	4.0E-03 (5.3E–03 to 4.7E–02)^b^	1.3E-4 (immature rat, sc) (1.0E-4) (immature rat, sc)^b^	NE (endogenous and exogenous, topical)
Genistein	2.1E-05 (1.2E–05 to 1.0E–02)^b^	1.2E-5 (immature rat, sc) (2.0E-4, 1.0E-5,1.7E-5) (immature rat, sc)^b^	NE (exogenous, oral) 70 mg/day x 1 year (50% daidzein, 30% glyceitin, and 20% genistein)

GL, Guideline; GL, Guideline-like; NE, No effect; NA, Not applicable.

^a^ Calculated by dividing the EC_50_ for E2 by the ERα agonist of interest.

^b^ Reproduced from Borgert et al. 2018 under a Creative Commons Attribution 4.0 International License (http://creativecommons.org/licenses/by/4.0/), with changes.

^c^ Calculated by dividing the EC_50_ for EE2 by the ERα agonist of interest.

^d^ Ip route considered equivalent to po route for calculation purposes due to first-pass metabolism (Al Shoyaib et al., 2019).

## 3 Results

### 3.1 ERTA and rodent uterotrophic data

EC_50_s from the ERTA and GLL uterotrophic studies are summarized in Table 2. For the OECD ERTA assays, relative potencies to E2 were concordant with the ranges specified in Borgert et al. (2018) for all ERα agonists but EE2 and genistein. For both of these, the relative potencies were within two-fold of the lower end of the potency range (Table 2). This difference is not considered biologically meaningful and could be due to the higher levels of endogenous ERβ in the VM7Luc ER TA assay. As such, the relative potencies of these ERα agonists to E2 in these OECD GL ERTA assays are considered concordant with Borgert et al. (2018) and to reproduce their results.

For the rodent uterotrophic data, results were expressed relative to EE2 since this is commonly used as a positive control and since GLL studies were identified for EE2 by both sc and po routes of exposure. In Kleinstreuer et al. (2016), the route of exposure (sc vs. po) and not species, strain, or the use of immature v. ovx animals was the greatest source of variability between studies. For E2, relative potency to EE2 was similar, and there were no data by Borgert et al. (2018) by which to determine concordance. For the rest of the ERα agonists, relative potency values were within five-fold or less of Borgert et al. (2018) for similar species and routes of exposure, with the exception of estrone. This was over 100-fold more potent relative to EE2 in our analysis than in Borgert et al. (2018). Different studies and routes of exposure were utilized (ip vs. sc), but these do not seem sufficient to explain the differences as sc would be expected to be more potent than ip, which retains first-pass metabolism. The difference is unclear. Even so, we consider the results of Borgert et al. (2018) to be largely concordant and are therefore reproduced.

### 3.2 Clinically observable endometrial effects

The EFSA ANS Panel (2015) concluded that isoflavone supplementation in peri-/menopausal women up to 30 months in doses up to 150 mg/day resulted in no changes in uterine thickening or histopathology but that non-malignant histopathological changes were observed at 60 months. We note that these changes were for 150 mg of isoflavones per day with five cases of statistically significant (p < 0.05) simple hyperplasia and one case of complex hyperplasia in 15 patients (6/154 or 4%) compared to 0/165 (or 0%) in controls (Unfer et al., 2004).

Histopathology is a more sensitive endpoint than endometrial thickening for detecting cancer and endometrial hyperplasia and is required by endometrial safety guidelines. The draft United States Food and Drug Administration (US FDA, 2003) and European Medicines Agency (European Medicines Agency, 2005) guidelines for endometrial safety studies for hormone replacement therapy (HRT) therefore both utilize histopathology and state that the rate of endometrial hyperplasia should not exceed 1% or 2%, respectively, for approximately 1 year of treatment. We further note that the European Medicines Agency guidelines provided power calculations for HRT that showed an incidence of hyperplasia of about 0.26% for 1 year of treatment in pooled studies and that power calculations indicated that a sample size of 300 patients would be required to achieve greater than 80% statistical power, which is the minimum required for an adequately powered study.

No studies cited by the EFSA ANS Panel (2015), include Unfer et al. (2004), satisfied the guideline criteria of a histopathological endpoint with at least 1 year of treatment duration and at least 300 subjects in the treatment group. Indeed, all studies that did have histopathological endpoints with at least 1 year of treatment cited by the EFSA ANS Panel would be underpowered by EMA guidelines (discussed above). Generally speaking, underpowered studies can have a higher propensity for false positives (Carnahan and Brown, 2024). Our literature search identified an additional study by Palacios et al. (2007) that was not mentioned by the EFSA ANS Panel that satisfied these criteria. This study is a multi-EU country endometrial safety study of Phytosoya (70 mg isoflavones per day comprised of 50% daidzein, 30% glyceitin, and 20% genistein) done according to EMA guidelines in 305 postmenopausal women for 1 year. This study was adequately powered and found no cases of endometrial hyperplasia. Vaginal bleeding was observed in 2% of subjects, all of whom had atrophic endometrium, with one case of severe metrorrhagia. The origin of the vaginal bleeding was unclear, but cancer and hyperplasia were ruled out. Palacios et al. (2007) concluded that Phytosoya may be a safe alternative to long-term hormone therapy.

E1 is produced endogenously, and exogenous E1 pharmaceuticals are available. A three-fold elevation in circulating E1 has been observed in postmenopausal people with a uterus with proliferative (Type 1) endometrial cancer vs. in healthy postmenopausal people. However, E1 is converted to E2 in endometrial tissue by the enzyme 17β-hydroxysteroid dehydrogenase, and inhibition of this enzyme has been considered for endometrial cancer therapeutics (Rižner et al., 2017). Therefore, the evidence indicates that E1-derived E2 via metabolic activity, and not E1 in the endometrium, is the driver of endometrial hyperplasia and cancer.

To attenuate hair loss from androgenic alopecia, 17α-E2 is used therapeutically when applied topically (Kim et al., 2012). It is also produced endogenously and has been identified in human urine (Tang et al., 2022). It is not associated with human endometrial hyperplasia or cancer.

### 3.3 Relative mechanistic potency evaluation

The relative mechanistic potencies of ERα agonists within ten-fold of E2 or EE2 positively predicted clinical effects in humans of endometrial hyperplasia and/or cancer. Less potent ERα agonists (100-fold less potent and beyond) negatively predicted clinical effects in humans (Table 2).

### 3.4 Inference performance evaluation (ACC’s SCF step 4)

Table 2 compares the relative mechanistic potencies (i.e., the ERαTA in combination with the rat uterotrophic assay) to clinically observe endometrial and cervical effects. Mechanistic potency predicts clinically observable effects (endometrial hyperplasia and/or endometrial/endocervical adenoma or carcinoma) within a ten-fold range of the synthetic agonist EE2. For weaker ERα agonists, the mechanistic potencies overpredict effects in humans.

### 3.5 Elements of ICCVAM’s SCF related to inference performance

Generally speaking, ACC’s SCF Step 4 informs specific elements within Steps 1 to 3 and 5 of ICCVAM’s SCF. By providing information on the extent to which the NAM’s response predicts the response of interest, ACC’s SCF Step 4 and the use of GL and GLL studies informs the following: context of use (Step 1), biological relevance (Step 2), relevance of performance standards and limits of use (Step 3), and transparency regarding NAM relevance to context of use (Step 5). Although not specifically included in Step 4 of ACC’s SCF, ICCVAM’s Step 4 (data integrity) is incorporated into Steps 3 and 5 of the ACC Scientific Confidence Framework, and ICVAM’s Step 6 (peer review) corresponds to Step 7 of the ACC Scientific Confidence Framework.

For the HRPT NAM in particular, its ability to predict uterine endometrial effects to include endometrial hyperplasia and adenomas and carcinomas (cancer) in humans informs the context of use (ICCVAM Step 1) by indicating that this NAM can function in several contexts of use, including screening/prioritization and hazard identification. It informs biological relevance (ICCVAM Step 2) by utilizing both established assays and relative functional potencies *in vitro* and *in vivo* in animals and bridges these to clinical data benchmarked with endogenous, pharmaceutical, or isoflavone compounds. Moreover, inherent in this NAM is the comparison of human clinical data to existing laboratory animal methods (the rat uterotrophic data in this instance).

For performance standards and the limits of use (Step 3), the use of GL and GLL studies in the construction of this NAM substantiated the adequate consideration of performance standards and limits of use.

## 4 Discussion

### 4.1 Clinically observable endometrial effects

Based on the only adequately powered study (Palacios et al., 2007) that was done in accordance with endometrial safety guidelines, we conclude that oral isoflavones (50% daidzein, 30% glycitein, and 20% genistein) up to 70 mg per day have no proliferative or carcinogenic effects on the endometrium.

### 4.2 HRPT

Responses in humans were seen only with substances with relative potencies for this NAM that were within 1 × 10^−1^. In other words, our results indicate that a HRPT of 1 × 10^−2^ or less would be protective in humans for the endpoints examined herein. This analysis also validates the results of Borgert et al. (2018), who concluded that an HRPT of 1 × 10^−4^ would be a conservative health-protective threshold. Recently, Borgert et al. (2024) demonstrated that the endogenous metabolome provides the physiological and biochemical basis of the HRPT. Using classical receptor occupancy models, they showed that a HRPT of 1 × 10^−4^ is conservative by a factor of at least 10, as they initially predicted. Those results agree with the analysis shown here. While it is beyond the scope of this paper to detail the many potential uses of this HRPT ERα agonist NAM, as indicated above, it could be used as a TTC or as a criterion for waiving further animal testing in a program such as the EDSP.

### 4.3 Inference performance evaluation (ACC’s SCF step 4)

The inference performance evaluation clearly demonstrated 1) that a substance having a relative mechanistic potency for ERα agonists within ten-fold of E2 or EE2 would be predicted, provided the magnitude, duration, and frequency of exposure were sufficient, to be capable of producing clinical effects in humans of endometrial hyperplasia and/or cancer, and 2) an HRPT of 1 × 10^−2^ or lower would be predicted not to produce such clinical effects in humans. This satisfies Step 4 of ACC’s SCF.

### 4.4 Elements of ICCVAM’s SCF related to inference performance

Several conditions of ICCVAM’s SCF can be satisfied using ACC’s inference performance as an initial screen. More conditions can be satisfied if GL and GLL studies are used in the construction or validation of the NAM. While this might seem counter-intuitive because SCFs can be thought of as a fit-for-purpose alternative to GL studies, it is nonetheless important to consider the level of the complexity of this particular NAM. Unlike simpler NAMs, the HRPT has embedded within it two sub-NAMs that each have test guidelines: the ERTA and the rodent uterotrophic assay. The HRPT also considers human clinical data to include endometrial safety studies on genistein, for which FDA and EMA guidelines exist. As such, it is relevant in the context of this particular NAM to consider the use of GL and GLL studies. We note that for consideration of other NAMs in SCFs, GL and GLL studies will likely not be available. That said, it was important to show herein that regulatory contexts that do commonly require GL studies and round-robin validations (such as in the OCED testing program) can be compatible with SCFs.

## 5 Conclusion

It is the shared responsibility of the regulatory science community to ensure that NAMs are fit-for-purpose for different applications and that SCFs provide a valuable framework for operationalization (Levine et al., 2025). While the concept of inference performance evaluation of an NAM seems to be implied within and across many of the elements in ICCVAM’s guidance (including biological relevance, reference compounds, comparison to existing laboratory animal methods, statistical model development, and data interpretation procedures), when implementing this guidance we recommend an explicit section be specifically devoted to analyzing inference performance of the NAM. It is critical to assess and transparently document the extent to which the NAM results or models predict the outcome of interest.

## Data Availability

The original contributions presented in the study are included in the article/Supplementary Material; further inquiries can be directed to the corresponding author.
